# A Loading Dose of Dexmedetomidine With Constant Infusion Inhibits Intraoperative Neuromonitoring During Thoracic Spinal Decompression Surgery: A Randomized Prospective Study

**DOI:** 10.3389/fphar.2022.840320

**Published:** 2022-03-07

**Authors:** Tun Liu, Yue Qin, Huaguang Qi, Zhenguo Luo, Liang Yan, Pengfei Yu, Buhuai Dong, Songchuan Zhao, Xucai Wu, Zhen Chang, Zhian Liu, Xuemei Liu, Tao Yuan, Houkun Li, Li Xiao, Gang Wang

**Affiliations:** ^1^ Department of Anesthesiology, Xi’an Honghui Hospital, Xi’an Jiaotong University Health Science Center, Xi’an, China; ^2^ Department of Functional Inspection Section, Xi’an Honghui Hospital, Xi’an Jiaotong University Health Science Center, Xi’an, China; ^3^ Department of Spine Surgery, Xi’an Honghui Hospital, Xi’an Jiaotong University Health Science Center, Xi’an, China; ^4^ Department of Gastrointestinal Surgery, Xijing Hospital of Digestive Diseases, Xijing Hospital, Fourth Military Medical University, Xi’an, China; ^5^ The Key Laboratory of Biomedical Information Engineering, Ministry of Education, School of Life Science and Technology, Institute of Biomedical Engineering, Xi’an Jiaotong University, Xi’an, China

**Keywords:** dexmedetomidine, intraoperative neuromonitoring, thoracic spinal decompression surgery, motor evoked potential (MEP), somatosensory evoked potential (SSEP)

## Abstract

**Background:** The effect of a bolus dose of dexmedetomidine on intraoperative neuromonitoring (IONM) parameters during spinal surgeries has been variably reported and remains a debated topic.

**Methods:** A randomized, double-blinded, placebo-controlled study was performed to assess the effect of dexmedetomidine (1 μg/kg in 10 min) followed by a constant infusion rate on IONM during thoracic spinal decompression surgery (TSDS). A total of 165 patients were enrolled and randomized into three groups. One group received propofol- and remifentanil-based total intravenous anesthesia (TIVA) (T group), one group received TIVA combined with dexmedetomidine at a constant infusion rate (0.5 μg kg^−1^ h^−1^) (D_1_ group), and one group received TIVA combined with dexmedetomidine delivered in a loading dose (1 μg kg^−1^ in 10 min) followed by a constant infusion rate (0.5 μg kg^−1^ h^−1^) (D_2_ group). The IONM data recorded before test drug administration was defined as the baseline value. We aimed at comparing the parameters of IONM.

**Results:** In the D_2_ group, within-group analysis showed suppressive effects on IONM parameters compared with baseline value after a bolus dose of dexmedetomidine. Furthermore, the D_2_ group also showed inhibitory effects on IONM recordings compared with both the D_1_ group and the T group, including a statistically significant decrease in SSEP amplitude and MEP amplitude, and an increase in SSEP latency. No significance was found in IONM parameters between the T group and the D_1_ group.

**Conclusion:** Dexmedetomidine delivered in a loading dose can significantly inhibit IONM parameters in TSDS. Special attention should be paid to the timing of a bolus dose of dexmedetomidine under IONM. However, dexmedetomidine delivered at a constant speed does not exert inhibitory effects on IONM data.

## 1 Introduction

Thoracic spinal decompression surgery (TSDS) is not as prevalent as cervical or lumbar spinal decompression surgery (Eggspuehler et al., 2007). Due to the rarity of thoracic spinal stenosis and the lack of adequate clinical experience (Stokes et al., 2019), TSDS confers a high risk for neurologic injury and even paralysis (Nuwer and Schrader, 2019). IONM is commonly used during spine surgery to provide real-time feedback of spinal neurological function. Early detection of neurologic dysfunction, using IONM, can alert the surgical team to initiate therapeutic interventions to limit or prevent further injury (Nuwer et al., 2012a; Melachuri et al., 2020). Previous studies reported that inhaled anesthetics are known to attenuate motor evoked potential (MEP) and somatosensory evoked potential (SSEP) as demonstrated by lower amplitude and increased latency on the waveforms (Zentner et al., 1976; Haghighi et al., 1990; Zentner et al., 1992; Chin Ted Chong et al., 2014). Furthermore, attenuated signals could erroneously be interpreted as neurologic injury or diminish the ability to appropriately monitor for neurologic injury (Nuwer et al., 2012b; Nuwer and Schrader, 2019; CoreyWalker and Park, 2020). Propofol has become one of the primary medications used for total intravenous anesthesia (TIVA) during spinal surgery with IONM (Macdonald et al., 2013; CoreyWalker and Park, 2020). Furthermore, remifentanil infusion offers the advantage of quicker recovery from anesthesia, which can facilitate the wake-up test (Imani et al., 2006), and has less variability in SSEP morphology (Samra et al., 2001). Moreover, it was demonstrated that propofol–remifentanil-based TIVA has an advantage over inhalation–intravenous combined anesthesia, because TIVA exerts less influence on synaptic transmission and has minimal effects on the amplitude and latency of IONM (Hermanns et al., 2007; CoreyWalker and Park, 2020). Those effects lead to a lower rate of false-positive waveform changes compared with inhaled anesthetics (Macdonald et al., 2013; CoreyWalker and Park, 2020). However, propofol had a dose-dependent inhibitory effect on MEP amplitude (Nathan et al., 2003; CoreyWalker and Park, 2020); the latest guideline recommended that propofol infusion rate <100 mg kg^−1^min^−1^ is the best recommendation under MEP monitoring (CoreyWalker and Park, 2020). So, maintaining lower propofol infusion rates by adding other types of intravenous anesthetics that do not adversely affect IONM signals can be beneficial.

The usage of dexmedetomidine in general anesthesia has both opioid-sparing (Nan Lin et al., 2019) and propofol-sparing (Ngwenyama et al., 2008) effects. So, dexmedetomidine has been increasingly used as an adjuvant to general anesthesia (Deiner et al., 2017; Silva-et al., 2019). However, effects of dexmedetomidine on SSEP and MEP remain a topic of hot debate (Endrit Bala et al., 2008; Tobias et al., 2008; Mahmoud et al., 2010; Rozet et al., 2015; Holt et al., 2020). Some authors demonstrated that dexmedetomidine does not influence IONM parameters when delivered by a loading dose and then followed by a constant infusion rate in adults (Lin et al., 2014; Rozet et al., 2015) and adolescents (Tobias et al., 2008). However, some authors demonstrated that dexmedetomidine administration can exert inhibitory effects on IONM (Mohamed Mahmoud et al., 2017; Holt et al., 2020). Moreover, dexmedetomidine enhances inhibitory synaptic transmission through activation of descending noradrenergic (NA) system (Yan Lu, 2007; Yusuke Funai a and Anthony, 2014). Furthermore, NA produces postsynaptic hyperpolarization (Grudt and Perl, 2002; Yan Lu, 2007). So, systemic administration of dexmedetomidine can therefore theoretically inhibit IONM to different degrees by enhancing inhibitory synaptic neurotransmission in both sensory and motor neurons.

We hypothesized that dexmedetomidine delivered in a loading dose (1 g kg^−1^ in 10 min) and then at a constant infusion rate (0.5 μg kg^−1^ h^−1^) has inhibitory effects on IONM recording. However, dexmedetomidine at a constant rate of infusion (0.5 μg kg^−1^ h^−1^) would not significantly impact IONM data. To test our hypothesis, we performed a randomized, double-blinded, placebo-controlled trial in adult patients who underwent TSDS in our hospital.

## 2 Methods

### 2.1 Ethics

Ethical approval for this study was provided by the Ethical Committee of Xi’an Honghui Hospital, Xi’an Jiaotong University Health Science Center, Xi’an, China, on October 1, 2018 (reference number No. 201801032) prior to patient enrolment and the start of the trial. The trial was registered at ChineseClinicalTrialRegistry.cn (Number: ChiCTR1800018685, October 3, 2018) prior to patient enrollment. Written informed consent was obtained from all subjects participating in the trial. This manuscript adheres to the applicable Consolidated Standards of Reporting Trials (CONSORT) guidelines.

### 2.2 Patients

**FIGURE 1:**
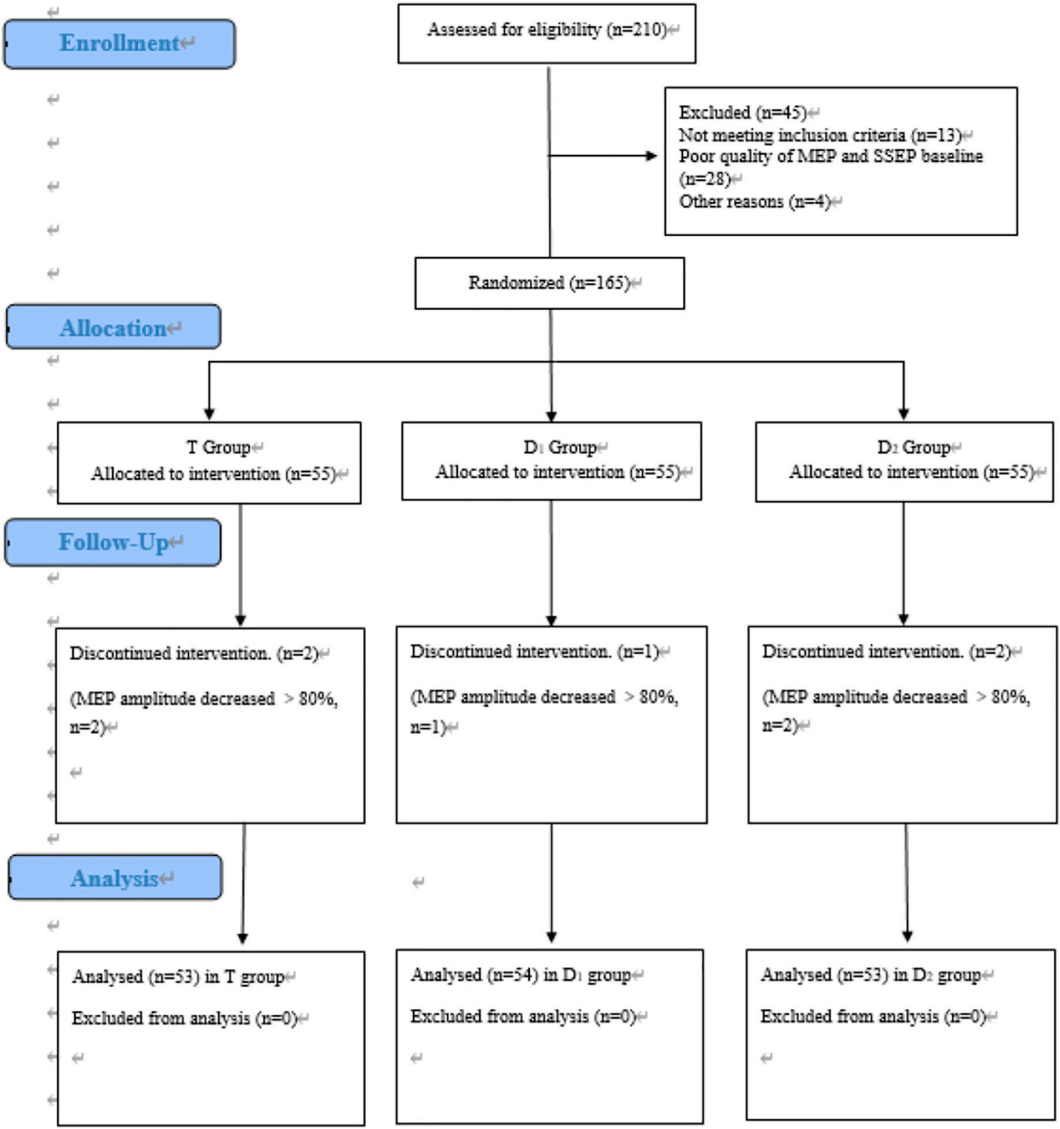
CONSORT flow diagram of patients’ inclusion. MEP, motor evoked potential. SSEP, somatosensory evoked potential. T group: propofol- and remifentanil-based total intravenous anesthesia (TIVA) group; D_1_ group: TIVA combined with dexmedetomidine at a constant infusion rate; D_2_ group: TIVA combined with dexmedetomidine delivered by a loading dose and then by a constant infusion rate.

A total of 210 patients were assessed for eligibility in our hospital. Inclusion criteria are as follows: (1) age between 18 and 60 years and ASA status from I to III; and (2) magnetic resonance image (MRI) studies showed thoracic spinal stenosis evidence (Tun Liu et al., 2021). Exclusion criteria are as follows: (1) poor quality of waveforms baseline; (2) patients who were unable to provide informed consent; (3) patients who were alcohol or drug abusers; and (4) not meeting inclusion criteria. After our inclusion and exclusion criteria were discussed, 165 patients identified as enrolled in the trial, and 160 patients finally completed the trial. Our study flowchart is shown in Figure 1. Anesthesia-related assessments were completed by an independent anesthesiologist in the post-anesthesia care unit (PACU); orthopedic-related assessments were completed by an independent orthopedic surgeon in the 6-month follow-ups.

### 2.3 Randomization and Blinding

Randomization was generated by SPSS v24.0 statistics software (IBM; Armonk, NY). The randomization results were concealed in sealed, prenumbered, opaque envelopes prepared by an independent bio-statistician. Those envelopes were kept in a box until required. From the start of muscle incision to muscle closure in the operation room, consecutively recruited patients were randomly assigned to receive an intravenous bolus of dexmedetomidine 1 μg kg^−1^ infusion over 10 min, then followed by continuous dexmedetomidine infusion at a rate of 0.5 μg kg^−1^ h^−1^ (the D_2_ group), or a volume matched bolus of 0.9% saline over 10 min, then followed by continuous dexmedetomidine infusion at a rate of 0.5 μg kg^−1^ h^−1^ (the D_1_ group), or a volume matched bolus and continuous infusion of 0.9% saline (the T group) in a 1:1:1 ratio, according to the random number allocation. An independent anesthesiologist was in charge of preparing and allocating the testing drugs to the corresponding anesthesiologists. An independent team of neurophysiologists was in charge of recording IONM data. So, the anesthesiologists, surgeons, neurophysiologists, and patients were blinded to treatment groups.

### 2.4 Anesthesia Protocol

Anesthesia induction was consistent with our previous protocol (Tun Liu et al., 2021). Anesthesia was induced by propofol 1.5–2.0 mg kg^−1^ and sufentanil 0.4–0.6 μg kg^−1^, midazolam 0.01 mg kg^−1^, and cisatracurium 0.10–0.15 mg kg^−1^. From tracheal intubation until surgical exposure, cisatracurium 1.5–2.5 mg kg^−1^ min^−1^ was maintained. A real-time train-of-four (TOF) ratio was recorded before eliciting MEP signals to rule out undesirable suppressive effects brought by muscle relaxants on IONM.

Anesthesia was maintained by the Diprifusor propofol infusion system, with a target-controlled infusion (TCI) of propofol 2.0–4.0 μg ml^−1^ and 0.15–0.30 μg kg^−1^ min^−1^. In the D_1_ group, dexmedetomidine was infused at 0.5 μg kg^−1^h^−1^ at a constant infusion rate from muscle incision to muscle closure. In the D_2_ group, dexmedetomidine was delivered by a loading dose (1.0 μg kg^−1^ over 10 min) and then followed by a constant infusion rate (0.5 μg kg^−1^ h^−1^). The depth of anesthesia was adjusted by varying the propofol or remifentanil doses based on bispectral monitor (BIS, Aspect Medical Systems Inc, United States ), and MAP was maintained between 70 and 80 mmHg and augmented by ephedrine as needed.

### 2.5 Acquisition of SSEP and MEP Signals

We recorded MEP to abductor hallucis (AH) muscles in the lower extremities and the first dorsal interosseous muscles in the upper extremities (control). Because previous studies demonstrated that AH muscles had the highest rate, even if the patients with preoperative severe motor deficit (Kobayashi et al., 1976a; Kobayashi et al., 1976b). The stimulation electrodes were inserted subcutaneously over motor cortex regions C3–C4 according to the 10/20 EEG international system. Recording electrodes are placed into the AH muscles and the first dorsal interosseous muscles. MEPs were elicited by subcutaneous needle electrodes by stimulating at a constant voltage ((220–360 V) and five to eight train pulses, with a duration of 300 μs. The signal analysis time was 100 m. The bandpass filter was between 10 and 1,500 Hz (Zhuang et al., 1976). The stimulations were delivered by a commercially available IONM stimulator (Cascade, Cadwell Laboratories Inc., United States) with responses recorded on the same device used for obtaining SSEP. The amplitudes of MEP were measured by recording baseline-to-first negative peak voltages.

We recorded SSEP to the median nerve for the upper extremity (control) and the posterior tibial nerve for the lower extremity. SSEPs were recorded using adhesive gel Ag-AgCl electrodes placed at Cz and Fpz positions for active and reference according to the 10/20 EEG international system. We performed median nerve stimulation bilaterally at the wrist, and performed posterior tibial nerve stimulation bilaterally at the head of the fibula or the medial malleolus of the ankle. The parameters of recording SSEP were as follows: the median nerve was stimulated at 15 mA, and the posterior tibial nerves were stimulated at 25 mA. The bandpass filter was between 30 and 300 Hz, and the waveforms were displayed in a 100-m window. The single pulse was set between 5.1 and 5.7 Hz. We measured the amplitude of P38-N45 and the latency of P38. To minimize signal interference, 300 to 400 stimulation repetition is averaged to obtain each SSEP sweep (Wang et al., 2017).

MEP peak-to-peak amplitudes, as well as amplitudes and latencies of SSEP obtained before administration of our testing drugs (dexmedetomidine or saline), were defined as baseline values. After administration of the testing drug, time course of the relative amplitude and relative latency of the evoked potentials in each group were calculated as follows: relative value (%) = absolute value/baseline value×100% (Furutani et al., 2019). Because absolute amplitudes of MEP differ greatly in patients, comparison of absolute amplitudes among different groups was very difficult (Furutani et al., 2019).

We adopted IONM warning criteria as our study drug discontinuation criteria, including the following: (1) a change in SSEP was defined as a decrease of greater than 50% in amplitude and/or 10% increase in latency of the baseline cortical wave, or as reported per each case; and (2) a change in MEP was defined as a decrease of more than 80% in amplitude of the baseline value, or as reported per case (Nuwer et al., 2012c; Nuwer and Schrader, 2019).

### 2.6 Time Points Set for Measuring IONM Parameters

MEP parameters were measured at seven time points: T_1_: Before dexmedetomidine or placebo infusion (at the same time as the start of muscle incision), we defined it as “baseline value”; T_2_: 10 min after dexmedetomidine or placebo infusion; T_3_: 20 min after dexmedetomidine or placebo infusion; T_4_: At the start of spine decompression; T_5_: 10 min after decompression; T_6_: 20 min after decompression; and T_7_: Muscle closure. SSEP parameters were measured at nine time points: T_1_: Before the start of dexmedetomidine or placebo infusion (at the same time as the start of muscle incision), we defined it as “baseline value”; T_2_: 5 min after dexmedetomidine infusion; T_3_: 10 min after infusion; T_4_: 15 min after infusion; T_5_: 20 min after infusion and then every 10 min until decompression; T_6_: At the start of spine decompression; T_7_: 5 min after spine decompression; T_8_: 10 min after spine decompression and then every 10 min until muscle closure; and T_9_: Surgery over.

### 2.7 Endpoints

The primary endpoint of the study was designed to evaluate the effects of dexmedetomidine by different approaches of administration on the amplitude and the latency of both SSEP and MEP in patients during TSDS.

Our secondary endpoints were aimed at evaluating the effects of dexmedetomidine on the intraoperative anesthetic requirement (consumption of propofol and remifentanil), hemodynamic stability (MAP and HR), anesthesia recovery time (time interval from cessation of anesthesia to following verbal commands and tracheal extubation) (Ku et al., 2002) in the operation room ,and postoperative pain scores assessed by VAS score in the PACU.

### 2.8 Sample Size Calculation and Statistical Analysis

Sample size calculation was performed by PASS 15 software (NCSS LLC, United States). Based on our pre-trial data (*n* = 24) on SSEP amplitude after 15 min of the test drug (dexmedetomidine or saline) infusion, SSEP amplitudes in the T, the D_1_, and the D_2_ groups were 1.83 ± 1.13 μV, 1.78 ± 1.61 μV, and 1.56 ± 1.90 μV, respectively. We chose the T and the D_1_ groups to calculate their sample size. Forty patients per group was the smallest sample size required to demonstrate a difference between the T group and the D_1_ group with an effect size of 0.8, a statistical power of 80%, an allocation ratio of the two groups of 1:1, and a two-sided *α* level of 0.05. Considering possible intraoperative waveform changes during the surgery, we planned to recruit at least 50 patients into each group.

All data were analyzed using SPSS24.0 statistics software (SPSS24.0, Chicago, IL, United States ). All measurement and enumeration data are presented as the mean± standard deviations (X± S D). The amplitude and latency of both MEP and SSEP were analyzed using the Mann–Whitney *U* test. Demographic data, hemodynamic parameters, anesthesia recovery time, and intraoperative anesthetic requirement were analyzed using the independent *t*-test among different groups. Within-group analysis was used. Qualitative or categorical variables were compared using the chi-square test or the Fisher test as appropriate. All reported *p* values less than 0.05 were considered to indicate statistical significance.

## 3 Results

Between October 2018 and December 2020, a total of 210 patients were assessed for eligibility, and 160 patients finally completed the trial. Figure 1 shows the flow diagram of the enrollment.

### 3.1 Comparison of the General Data of the Study Population

Compared with the T group, patients in the D_1_ group (591.9 ± 102.5 vs. 787.8 ± 68.3, *p* < 0.05) and the D_2_ group (539.4 ± 70.1 vs. 787.8 ± 68.3, *p* < 0.05) showed much less propofol consumption. Furthermore, patients in the D_1_ group (2,512.5 ± 280.4 vs. 2,981.9 ± 465.8, *p* < 0.05) and the D_2_ group (2,315.5 ± 338.5 vs. 2,981.9 ± 465.8, *p* < 0.05) also showed much less remifentanil consumption. Moreover, patients in the D_2_ group showed more ephedrine consumption than those in the D_1_ group (20.1 ± 8.6 vs. 11.1 ± 8.1, *p* < 0.05) and the T group (20.1 ± 8.6 vs. 9.6 ± 7.0, *p* < 0.05). Furthermore, the D_2_ group showed a longer anesthesia recovery time, compared with the D_1_ group (15.7 ± 4.1 vs. 12.6 ± 2.8, *p* < 0.05) and the T group (15.7 ± 4.1 vs. 14.9 ± 3.7, *p* < 0.05). Although the differences have statistical significance, they are in the order of 2 or 3 min, which is not clinically relevant. No significance was found among the different groups in terms of age, sex, weight, height, symptom duration, operation time, bleeding volume, surgical location, MAP, or heart rate (HR) before the start of anesthesia induction, and VAS score after general anesthesia recovery in the PACU. As depicted in Table 1. Furthermore, bleeding volume in our study was 569.15 ± 217.30 ml. In particular, massive blood loss in a short time (>500 ml in less than 30 min) during the decompression procedure was observed in 31 patients.

**TABLE 1:** The general data of the three groups.

	T group (*n* = 53)	D_1_ group (*n* = 54)	D_2_ group (*n* = 53)
Demographic data			
Age (years)	42.3 ± 16.2	43.4 ± 15.3	42.9 ± 15.7
Sex (M/F)	30/23	29/25	31/23
Height (cm)	168.7 ± 6.2	168.1 ± 5.6	169.2 ± 6.4
Weight (kg)	60.2 ± 12.8	61.2 ± 11.3	61.9 ± 10.7
Symptom duration (months)	4.7 ± 5.3	5.0 ± 4.6	4.9 ± 4.1
Perioperative data			
Operation time (min)	212.3 ± 105.1	209.0 ± 99.7	204.6 ± 101.2
Bleeding volume (ml)	563.2 ± 213.1	571.2 ± 200.2	559.2 ± 220.4
MAP before anesthesia induction (mmHg)	74.3 ± 5.5	74.8 ± 6.7	73.9 ± 6.3
HR before anesthesia induction (bpm)	75.2 ± 5.1	75.7 ± 6.3	75.4 ± 6.8
Surgical location			
T_1-8_ (*n* = )	7	6	6
T_9-12_ (*n* = )	46	48	47
VAS score in PACU	4.6 ± 1.4	4.9 ± 0.8	4.8 ± 1.1
Anaesthesia recovery time (min)	14.9 ± 3.7	12.6 ± 2.8^a^	15.7 ± 4.1^c^
Propofol consumption (mg)	787.8 ± 68.3	591.9 ± 102.5^a^	539.4 ± 70.1^b^ ^,^ ^c^
Remifentanil consumption (ug)	2,981.9 ± 465.8	2,512.5 ± 280.4^a^	2,315.5 ± 338.3^b^ ^,^ ^c^
Ephedrine consumption (mg)	9.6 ± 7.0	11.1 ± 8.1	20.1 ± 8.6^b^ ^,c^
IONM baseline value			
SSEP amplitude (μV)	1.87 ± 1.05	1.91 ± 1.11	1.86 ± 1.08
SSEP latency (ms)	42.81 ± 3.94	43.12 ± 3.36	42.98 ± 3.40

VAS: visual analogue scale; PACU: post anesthesia care unit. IONM: intraoperative neuromonitoring; SSEP: somatosensory evoked potential; MEP: motor evoked potential; T group: propofol- and remifentanil-based total intravenous anesthesia group; D_1_ group: TIVA, combined with dexmedetomidine at a constant infusion rate; D_2_ group: TIVA, combined with dexmedetomidine delivered by loading dose and then by a constant infusion rate. Data were expressed as mean ± standard deviations (X±SD) for VAS, score, time of anesthesia recovery, propofol consumption, remifentanil consumption, and ephedrine consumption. The amplitude and latency of both MEP and SSEP were analyzed using the Mann–Whitney *U* test. Demographic data, hemodynamic parameters, anesthesia recovery time, and intraoperative anesthetic requirement were analyzed using the independent *t*-test among different groups. Qualitative or categorical variables were compared using the chi-square test or the Fisher test as appropriate.

a *p*<0.05, D_1_ group compared with the T group.

b *p*<0.05, D_2_ group compared with the T group.

c *p*<0.05, D_2_ group compared with the D_1_ group.

### 3.2 Comparison of SSEP and MEP Parameters

#### 3.2 1 Comparison of the IONM Baseline Values Among Different Groups

No significance was found in both amplitude and latency before the start of dexmedetomidine or placebo infusion among different groups. SSEP amplitude baseline values in the T, the D_1_, and the D_2_ groups were 1.87 ± 1.05 μV, 1.91 ± 1.11 μV, and 1.86 ± 1.08 μV, respectively. SSEP latency baseline value in the T, the D_1_, and the D_2_ groups were 42.81 ± 3.94 m, 43.12 ± 3.36 m, and 42.98 ± 3.40 m, respectively.

#### 3.2.2 Comparison of Time Course of the Relative Amplitude and Relative Latency of the Evoked Potentials

**FIGURE 2:**
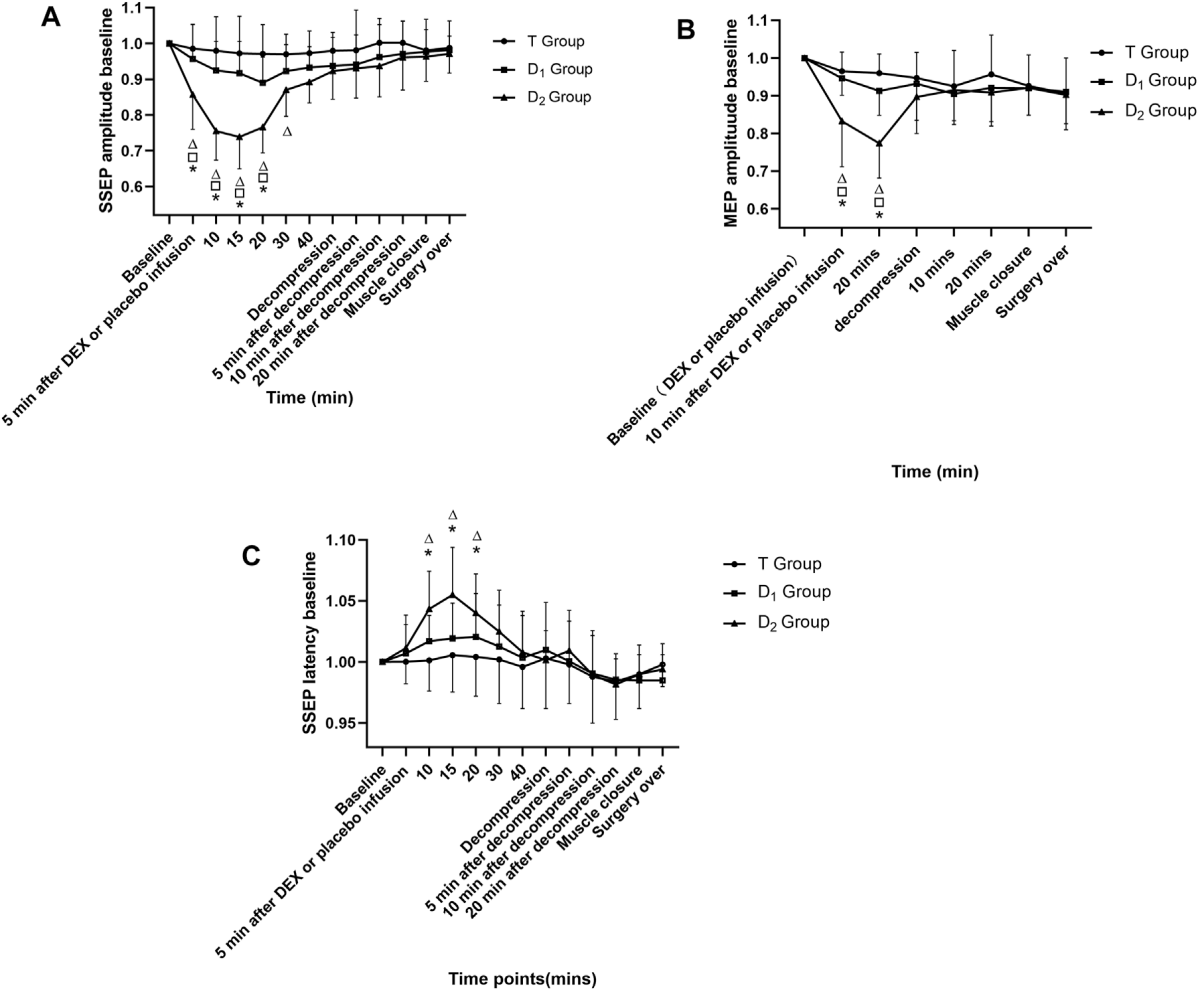
Time course of SSEP and MEP amplitude variability, as well as SSEP latency variability in the three groups. ^△^Compared with baseline value (after muscle incision, but before dexmedetomidine or placebo infusion) at corresponding time points, *p*<0.05; compared with the D_1_ group at corresponding time points, *p*<0.05; * compared with the T group at corresponding time points, *p*<0.05. DEX: dexmedetomidine; the T group: propofol- and remifentanil-based total intravenous anesthesia group; the D_1_ group: TIVA combined with dexmedetomidine at a constant infusion rate; the D_2_ group: TIVA combined with dexmedetomidine delivered by a loading dose and then by a constant infusion rate.

In the D_2_ group, within-group analysis showed suppressive effects on IONM parameters compared with baseline value after dexmedetomidine (1 μg kg^−1^ in 10 min) infusion, including a significant decrease in SSEP amplitude (lasted for 25 min) and MEP amplitude (lasted for at least 10 min), and an increase in SSEP latency (lasted for 10 min). Compared with the D_1_ group and the T group, the D_2_ group also showed inhibitory effects on IONM recordings, including a significantly lower SSEP amplitude (lasted for 15 min) and MEP amplitude (lasted for at least 10 min) and a significantly prolonged SSEP latency (lasted for 10 min). No significance was found in IONM data between the T group and the D_1_ group, as depicted in Figure 2. Furthermore, in the D_2_ group, within-group analysis showed that a bolus of dexmedetomidine (1 μg kg^−1^ in 10 min) could increase SSEP latency by 5.50% ± 3.51%, and decreased MEP amplitude and SSEP amplitude by 27.13% ± 12.30% and 24.75% ± 15.04%, respectively, compared with the baseline value.

### 3.3 Comparison of MAP and HR Parameters

**FIGURE 3:**
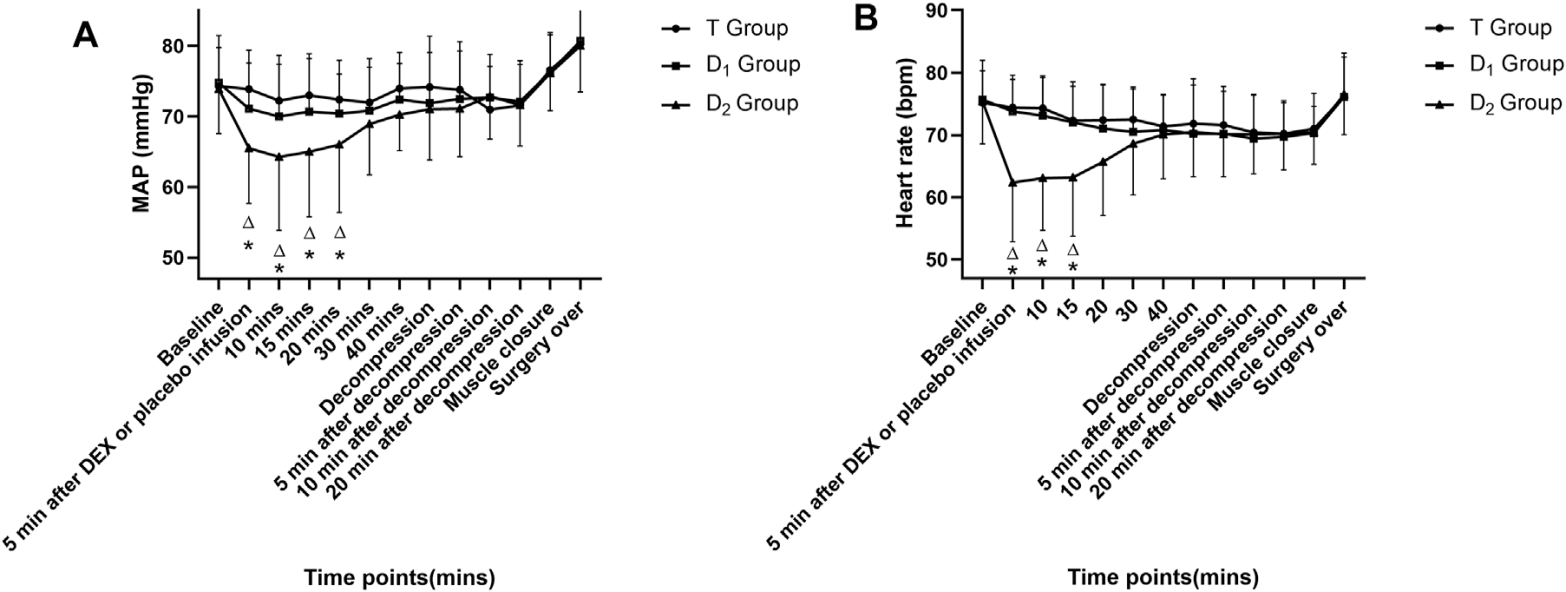
Time course of intraoperative mean arterial pressure (MAP) (as depicted in 3A) and heart rate (HR) (as depicted in 3B) in the three groups. ^△^ Compared with baseline value (after muscle incision, but before dexmedetomidine or placebo infusion) at corresponding time points, *p*<0.05; * compared with the T group at corresponding time points, *p*<0.05. DEX: dexmedetomidine; the T group: propofol- and remifentanil-based total intravenous anesthesia group; the D_1_ group: TIVA combined with dexmedetomidine at a constant infusion rate; the D_2_ group: TIVA combined with dexmedetomidine delivered by a loading dose and then by a constant infusion rate.

No significance was found in MAP and HR before the start of dexmedetomidine or placebo infusion among different groups (Table 1). In the D_2_ group, within-group analysis showed a significant decrease in MAP and HR compared with baseline after dexmedetomidine (1 μg kg^−1^ in 10 min) infusion and lasted for 15 min. Furthermore, a significant decrease in the MAP and HR was found between the T group and the D_2_ group after a bolus dose of dexmedetomidine. As is depicted in Figure 3. This indicates an unstable cardiovascular system after dexmedetomidine was delivered by a bolus dose. In contrast, after the testing drug was delivered only at a constant infusion rate in the D_1_ group, no significant difference in MAP or HR at various time points was found, and both parameters were maintained within the clinically normal range.

## 4 Discussion

Our study aimed at exploring effects of dexmedetomidine by different approaches of administration on the amplitude and the latency of both SSEP and MEP in patients during TSDS. The T group received TIVA, the D_1_ group received TIVA combined with a constant infusion rate of dexmedetomidine, and the D_2_ group received TIVA combined with dexmedetomidine delivered in a loading dose and then at a constant infusion rate. Within-group analysis showed that dexmedetomidine in the D_2_ group exerted inhibitory effects on amplitude of both SSEP and MEP, as well as latency of SSEP. Compared with both the T group and the D_1_ group, patients in the D_2_ group also showed a significant decrease in amplitude of the waveforms and an increase in SSEP latency. Here, we demonstrated that dexmedetomidine delivered in a loading dose and then at a constant infusion rate had inhibitory effects on IONM recording in TSDS. However, dexmedetomidine delivered only at a constant infusion rate did not influence IONM parameters.

### 4.1 Time Points Set for Measuring IONM Parameters

Rozet et al. (2015) demonstrated that dexmedetomidine delivery by a bolus dose (0.6 μg kg^−1^ infused in 10 min) and followed by 0.6 μg kg^−1^ h^−1^ did not affect SSEP and MEP in adult patients during spine surgery. However, Rozet recorded both amplitude and latency twice within the first 30 min after dexmedetomidine administration and then recorded IONM parameters every 30 min until 240 min after dexmedetomidine administration. Statistically significant change in IONM parameters would likely be missed and ignored, because based on our results, compared with the T group and the D_1_ group, the significant inhibitory effects of dexmedetomidine delivered in a bolus dose on SSEP amplitude, SSEP latency, and MEP amplitude lasted for 15 min, 10 min, and at least 10 min, respectively. If the interval of IONM recording is more than 15 min within the first 30 min after dexmedetomidine (1 μg kg^−1^), it would likely miss the statistically significant change in amplitude and latency of evoked potentials.

Furutani et al. obtained MEP waveforms at 2, 4, 6, 8, and 10 min after a bolus dose of the testing drug (Furutani et al., 2019). However, we limited the frequency of MEP recording. First, MEP recording has more possibilities of adverse events (Yoshida et al., 1976a). Second, according to the latest guidance (Nuwer et al., 2012a) and our previous study (Tun Liu et al., 2021), it was recommended that every high-risk procedure (Yoshida et al., 1976b) (posterior decompression in OPLL patients and correction in deformity patients) after 10–20 min (Tun Liu et al., 2021), which is a critical time point, needs to put special emphasis on the change of IONM. Therefore, we recorded IONM parameters before and after high-risk procedures or according to specific needs during surgery to judge whether patients have spinal injuries.

### 4.2 Effects of Dexmedetomidine on Both Amplitude and Latency

Yusuke Funai and Anthony (2014) demonstrated that dexmedetomidine infusion enhances inhibitory synaptic transmission in the superficial dorsal horn (SDH) by activating the descending noradrenergic (NA) system. Then, NA can produce postsynaptic hyperpolarization (Grudt and Perl, 2002; Yan Lu, 2007). So, systemic administration of dexmedetomidine can therefore theoretically inhibit IONM parameters to different degrees by enhancing inhibitory synaptic neurotransmission in both motor and sensory neurons, especially when dexmedetomidine was delivered by a bolus dose (1 μg kg^−1^ in 10 min), because larger doses of dexmedetomidine can lead to obvious inhibitory synaptic neurotransmission in neurons, thus resulting in attenuated amplitude and latency of the evoked potentials (Mohamed Mahmoud et al., 2010; Chen et al., 2015). Hence, IONM parameters would be more likely to be inhibited after dexmedetomidine 1 μg kg^−1^ in 10 min. On the contrary, IONM waveforms would be less likely to be affected when smaller doses of dexmedetomidine were administered (at a constant infusion rate, 0.5 μg kg^−1^ h^−1^) (Endrit Bala et al., 2008; Chen et al., 2015; Li et al., 2016; Liu et al., 2021).

Mahmoud et al. (2010) and Mohamed Mahmoud et al. (2017) reported that dexmedetomidine delivered by loading dose and then a constant infusion rate added to TIVA can decrease MEP amplitude in patients aged between 10 and 25 years. We excluded patients aged under 18 years old and abandon inhalation anesthetics in our study, for two reasons: (1) dexmedetomidine is not recommended for adolescents according to the FDA (SudIvya Sharma, 2013); and (2) accuracy of IONM could be adversely affected by the immature neural pathways of adolescents, and the differences between adolescents and adults in neuron structure and organization can increase the bias to the conclusion^26^. Although we exclude adolescent patients, we also reached a similar conclusion: dexmedetomidine (1 μg kg^−1^ in 10–20 min) does exert statistically inhibitory effects on IONM parameters. In our study, dexmedetomidine (1 μg kg^−1^ in 10 min) can inhibit IONM parameters within warning criteria (Nuwer and Schrader, 2019; Tun Liu et al., 2021). The suppressive effects on MEP and SSEP lasted for more than 10 min, and no more than 25 min, respectively. Therefore, we recommended that anesthesiologists should consider the time point of a bolus dose of dexmedetomidine administration during spine surgery.

### 4.3 Effects of Dexmedetomidine on Cardiovascular Stability

MAP could influence human autoregulation by maintaining stable cerebral blood flow (CBF) and spinal cord blood flow (SCBF) (Crystal et al., 2014; Meng et al., 2019). Furthermore, previous studies revealed that hypotension intraoperatively can increase the likelihood of neurologic deficits by reducing spinal cord perfusion pressure (Joshua Yang et al., 2018; CoreyWalker and Park, 2020). Schwan et al. (2020) reported that evoked potential waveforms can be lost after recurrent bradycardia during spinal surgery. So, bradycardia and hypotension should be avoided strictly during spinal surgeries, according to the latest guidance (Vitale et al., 2014; CoreyWalker and Park, 2020). A loading dose of dexmedetomidine (1 μg/kg in 10 min) has inhibitory effects on both MAP and HR, and lasted for 15 min. Anesthesiologists and neurophysiologists should be aware of this effect.

Lieberman et al. demonstrated that the serum concentration of propofol may increase dramatically during hemorrhage in a swine model (JeremyLieberman, 2013; Lieberman et al., 2018a; Lieberman et al., 2018b). Furthermore, hemorrhage is associated with a decrease in MEP amplitude (Lieberman et al., 2018b). Furthermore, elevated levels of propofol infusion can occasionally lead to hyperlactacidemia (Parke et al., 1992; Marinella, 1996). Therefore, to avoid excessive propofol consumption, using an adjuvant in general anesthesia that does not have an adverse influence on IONM and cardiovascular stability can be beneficial. We demonstrated that dexmedetomidine administrated at a constant infusion rate does not influence IONM or cardiovascular stability and has propofol-sparing effects.

### 4.4 Limitations

Our study has some limitations. Firstly, a decrease in MAP and HR after dexmedetomidine (1 μg kg^−1^) could be identified by the attending anesthesiologists and might confound our blinding. However, it is less likely to affect our results, because the IONM parameters were recorded by an independent and blinded neurophysiologist. Secondly, we do not have MEP waveforms 20 min after dexmedetomidine (1 μg kg^−1^) until decompression, because we limited the frequency of MEP recording. So, the inhibitory effects of dexmedetomidine (1 μg kg^−1^ in 10 min) on MEP amplitude might last for more than 10 min. However, there is no significant difference in MEP amplitude among different groups at the start of spine decompression, indicating adequate time to eliminate the adverse effects of dexmedetomidine on MEP amplitude. So, the inhibitory effects of dexmedetomidine (1 μg kg^−1^) were less likely to affect our MEP waveforms before and after high-risk procedures.

## 5 Conclusion

A bolus dose of dexmedetomidine with a constant infusion rate can significantly increase the latency of SSEP and reduce the amplitude of both SSEP and MEP in TSDS. Special attention should be paid to the timing of dexmedetomidine (1 μg kg^−1^ in 10 min) administration under IONM. However, dexmedetomidine can be delivered at a constant rate (0.5 μg kg^−1^ h^−1^) because it does not exert an inhibitory effect on IONM parameters.

## Data Availability

The original contributions presented in the study are included in the article/Supplementary Material, further inquiries can be directed to the corresponding author.

## References

[B1] Chen Z. Lin S. Shao W. (2015). Effects on Somatosensory and Motor Evoked Potentials of Senile Patients Using Different Doses of Dexmedetomidine during Spine Surgery. Irish J. Med. Sci. 184, 813–818. 10.1007/s11845-014-1178-0 25183287

[B2] Chin Ted Chong M. Anaes M. M Fanzca Manninen P. Frcpc M. D. Vanitha Sivanaser M. (2014). Direct Comparison of the Effect of Desflurane and Sevoflurane on Intraoperative Motor-Evoked Potentials Monitoring. J. Neurosurg. Anesthesiol 26, 306–312. 10.1097/ANA.0000000000000041 24487732

[B3] CoreyWalker H. J. K. T. Park P. (2020). Neuroanesthesia Guidelines for Optimizing Transcranial Motor Evoked Potentials Neuromonitoring during Deformity and Complex Spinal Surgery: A Delphi Consensus Study. SPINE. 10.1097/BRS.0000000000003433 32539292

[B4] Crystal G. J. Czinn E. A. Salem M. R. (2014). The Mechanism of Increased Blood Flow in the Brain and Spinal Cord during Hemodilution. Anesth. Analg 118, 637–643. 10.1213/ANE.0000000000000078 24557108

[B5] Deiner S. Luo X. Lin H. M. Sessler D. I. Saager L. Sieber F. E. (2017). Intraoperative Infusion of Dexmedetomidine for Prevention of Postoperative Delirium and Cognitive Dysfunction in Elderly Patients Undergoing Major Elective Noncardiac Surgery: A Randomized Clinical Trial. JAMA Surg. 152, e171505. 10.1001/jamasurg.2017.1505 28593326 PMC5831461

[B6] Eggspuehler A. Sutter M. A. Grob D. Porchet F. Jeszenszky D. Dvorak J. (2007). Multimodal Intraoperative Monitoring (MIOM) during Surgical Decompression of Thoracic Spinal Stenosis in 36 Patients. Eur. Spine J. 16 (Suppl. 2), S216–S220. 10.1007/s00586-007-0425-8 17610089 PMC2072894

[B7] Endrit Bala D. I. S. Nair D. R. McLain R. Dalton J. E. (2008). Ehab Farag, Motor and Somatosensory Evoked Potentials Are Well Maintained in Patients Given Dexmedetomidine during Spine Surgery. Anesthesiology 109, 417–425. 10.1097/ALN.0b013e318182a467 18719439

[B8] Furutani K. Deguchi H. Matsuhashi M. Mitsuma Y. Kamiya Y. Baba H. (2019). A Bolus Dose of Ketamine Reduces the Amplitude of the Transcranial Electrical Motor-Evoked Potential. J. Neurosurg. Anesthesiology Publish Ahead Print. 10.1097/ana.0000000000000653 31633576

[B9] Grudt T. J. Perl E. R. (2002). Correlations between Neuronal Morphology and Electrophysiological Features in the Rodent Superficial Dorsal Horn. J. Physiol. 540, 189–207. 10.1113/jphysiol.2001.012890 11927679 PMC2290200

[B10] Haghighi S. S. Green K. D. Oro J. J. Drake R. K. Kracke G. R. (1990). Depressive Effect of Isoflurane Anesthesia on Motor Evoked Potentials. Neurosurgery 26, 993–997. 10.1097/00006123-199006000-00012 2362677

[B11] Hermanns H. Lipfert P. Meier S. Jetzek-Zader M. Krauspe R. Stevens M. F. (2007). Cortical Somatosensory-Evoked Potentials during Spine Surgery in Patients with Neuromuscular and Idiopathic Scoliosis under Propofol-Remifentanil Anaesthesia. Br. J. Anaesth. 98, 362–365. 10.1093/bja/ael365 17237215

[B12] Holt F. Strantzas S. Zaarour C. Chamlati R. Vreugdenhil I. Luginbuehl I. (2020). The Effect of Dexmedetomidine on Motor-Evoked Potentials during Pediatric Posterior Spinal Fusion Surgery: a Retrospective Case-Control Study. Can. J. Anaesth. 10.1007/s12630-020-01758-6 32700209

[B13] Imani F. Jafarian A. Hassani V. Khan Z. H. (2006). Propofol-alfentanil vs Propofol-Remifentanil for Posterior Spinal Fusion Including Wake-Up Test. Br. J. Anaesth. 96, 583–586. 10.1093/bja/ael075 16567343

[B14] JeremyLieberman J. F. A. (2013). Russ Lyon, Mark D. Rollins, Effect of Hemorrhage and Hypotension on Transcranial Motor-Evoked Potentials in Swine. Anesthesiology 119, 1109–1119. 10.1097/ALN.0b013e31829d4a92 23770600

[B15] Joshua Yang B. Skaggs D. L. Mmm M. D. Priscella Chan M. S. Suken A. Shah M. D. (2018). Raising Mean Arterial Pressure Alone Restores 20% of Intraoperative Neuromonitoring Losses. SPINE 43, 890–894. 10.1097/BRS.0000000000002461 29049087

[B16] Kobayashi K. Imagama S. Ando K. Yoshida G. Ando M. Kawabata S. (1976a). Characteristics of Cases with Poor Transcranial Motor-Evoked Potentials Baseline Waveform Derivation in Spine Surgery: A Prospective Multicenter Study of the Monitoring Committee of the Japanese Society for Spine Surgery and Related Research. Spine (Phila Pa. 46, E1211–E1219. 10.1097/BRS.0000000000004074 34714796

[B17] Kobayashi K. Imagama S. Yoshida G. Ando M. Kawabata S. Yamada K. (1976b). Effects of Preoperative Motor Status on Intraoperative Motor-Evoked Potential Monitoring for High-Risk Spinal Surgery: A Prospective Multicenter Study. Spine (Phila Pa. 46, E694–E700. 10.1097/BRS.0000000000003994 34027929

[B18] Ku A. S. Hu Y. Irwin M. G. Chow B. Gunawardene S. Tan E. E. (2002). Effect of Sevoflurane/nitrous Oxide versus Propofol Anaesthesia on Somatosensory Evoked Potential Monitoring of the Spinal Cord during Surgery to Correct Scoliosis. Br. J. Anaesth. 88, 502–507. 10.1093/bja/88.4.502 12066725

[B19] Li Y. Meng L. Peng Y. Qiao H. Guo L. Han R. (2016). Effects of Dexmedetomidine on Motor- and Somatosensory-Evoked Potentials in Patients with Thoracic Spinal Cord Tumor: a Randomized Controlled Trial. BMC Anesthesiol 16, 51. 10.1186/s12871-016-0217-y 27484701 PMC4970285

[B20] Lieberman J. A. Feiner J. Rollins M. Lyon R. Jasiukaitis P. (2018). Changes in Transcranial Motor Evoked Potentials during Hemorrhage Are Associated with Increased Serum Propofol Concentrations. J. Clin. Monit. Comput. 32, 541–548. 10.1007/s10877-017-0057-4 28856576

[B21] Lieberman J. A. Feiner J. Rollins M. Lyon R. Jasiukaitis P. (2018). Correction to: Changes in Transcranial Motor Evoked Potentials during Hemorrhage Are Associated with Increased Serum Propofol Concentrations. J. Clin. Monit. Comput. 32, 581. 10.1007/s10877-017-0075-2 28856576

[B22] Lin S. Dai N. Cheng Z. Shao W. Fu Z. (2014). Effect of Dexmedetomidine-Etomidate-Fentanyl Combined Anesthesia on Somatosensory- and Motor-Evoked Potentials in Patients Undergoing Spinal Surgery. Exp. Ther. Med. 7, 1383–1387. 10.3892/etm.2014.1555 24940443 PMC3991509

[B23] Liu X. Li Y. Kang L. Wang Q. (2021). Recent Advances in the Clinical Value and Potential of Dexmedetomidine. J. Inflamm. Res. 14, 7507–7527. 10.2147/JIR.S346089 35002284 PMC8724687

[B24] Macdonald D. B. Skinner S. Shils J. Yingling C. (2013). Intraoperative Motor Evoked Potential Monitoring - a Position Statement by the American Society of Neurophysiological Monitoring. Clin. Neurophysiol. 124, 2291–2316. 10.1016/j.clinph.2013.07.025 24055297

[B25] Mahmoud M. Sadhasivam S. Salisbury S. Nick T. G. Schnell B. Sestokas A. K. (2010). Susceptibility of Transcranial Electric Motor-Evoked Potentials to Varying Targeted Blood Levels of Dexmedetomidine during Spine Surgery. Anesthesiology 112, 1364–1373. 10.1097/ALN.0b013e3181d74f55 20460997

[B26] Marinella M. A. (1996). Lactic Acidosis Associated with Propofol. Chest 109, 292. 10.1378/chest.109.1.292 8549205

[B27] Melachuri S. R. Stopera C. Melachuri M. K. Anetakis K. Crammond D. J. Castellano J. F. (2020). The Efficacy of Somatosensory Evoked Potentials in Evaluating New Neurological Deficits after Spinal Thoracic Fusion and Decompression. J. Neurosurg. Spine, 1–6. 10.3171/2019.12.SPINE191157 32114528

[B28] Meng L. Wang Y. Zhang L. McDonagh D. L. (2019). Heterogeneity and Variability in Pressure Autoregulation of Organ Blood Flow: Lessons Learned over 100+ Years. Crit. Care Med. 47, 436–448. 10.1097/CCM.0000000000003569 30516567

[B29] Mohamed Mahmoud M. D. Sadhasivam S. Sestokas A. K. Paul Samuels M. D. John McAuliffe M. D. (2017). Loss of Transcranial Electric Motor Evoked Potentials during Pediatric Spine Surgery with Dexmedetomidine. Anesthesiology 106, 393–396. 10.1097/00000542-200702000-00027 17264733

[B30] Mohamed Mahmoud S. S. Salisbury S. Nick T. G. (2010). Beverly Schnell, Anthony K. Sestokas, Cheryl Wiggins, Au. Paul Samuels, Thomas Kabalin, John McAuliffe., Susceptibility of Transcranial Electric Motor-Evoked Potentials to Varying Targeted Blood Levels of Dexmedetomidine during Spine Surgery. Anesthesiology, 1364–1373. 10.1097/ALN.0b013e3181d74f55 20460997

[B31] Nan Lin M. Vutskits L. Bebawy J. F. Gelb A. W. (2019). Perspectives on Dexmedetomidine Use for Neurosurgical Patients. J. Neurosurg. Anesthesiol 31, 366–377. 10.1097/ANA.0000000000000554 30363004

[B32] Nathan N. Tabaraud F. Lacroix F. Moulies D. Viviand X. Lansade A. (2003). Influence of Propofol Concentrations on Multipulse Transcranial Motor Evoked Potentials. Br. J. Anaesth. 91, 493–497. 10.1093/bja/aeg211 14504148

[B33] Ngwenyama N. E. Anderson J. Hoernschemeyer D. G. Tobias J. D. (2008). Effects of Dexmedetomidine on Propofol and Remifentanil Infusion Rates during Total Intravenous Anesthesia for Spine Surgery in Adolescents. Pediatric Anesthesia. 10.1111/j.1460-9592.2008.02787.x 19076573

[B34] Nuwer M. R. Emerson R. G. Galloway G. Legatt A. D. F. A. A. N. Lopez J. Minahan R. (2012a). Evidence-based Guideline Update: Intraoperative Spinal Monitoring with Somatosensory and Transcranial Electrical Motor Evoked Potentials. Neurology, 585–589. 10.1097/WNP.0b013e31824a397e 22353994

[B35] Nuwer M. R. Schrader L. M. (2019). Spinal Cord Monitoring. Handb Clin. Neurol. 160, 329–344. 10.1016/B978-0-444-64032-1.00021-7 31277858

[B36] Nuwer M. R. Emerson R. G. Galloway G. Legatt A. D. Lopez J. Minahan R. (2012c). Evidence-based Guideline Update: Intraoperative Spinal Monitoring with Somatosensory and Transcranial Electrical Motor Evoked Potentials. J. Clin. Neurophysiol. 29, 101–108. 10.1097/WNP.0b013e31824a397e 22353994

[B37] Nuwer M. R. Emerson R. G. Galloway G. Legatt A. D. Lopez J. Minahan R. (2012b). Therapeutics, N. Technology Assessment Subcommittee of the American Academy of, and S. American Clinical Neurophysiology, Evidence-Based Guideline Update: Intraoperative Spinal Monitoring with Somatosensory and Transcranial Electrical Motor Evoked Potentials: Report of the Therapeutics and Technology Assessment Subcommittee of the American Academy of Neurology and the American Clinical Neurophysiology Society. Neurology 78, 585–589. 10.1212/WNL.0b013e318247fa0e 22351796

[B38] Parke J. E. S. T. J. Rice A. S. C. Greenaway C. L. Bray R. J. Smith P. J. Waldmann C. S. (1992). Metabolic Acidosis and Fatal Myocardial Failure after Propofol Infusion in Children: Five Case Reports. BMJ 305, 613–616. 10.1136/bmj.305.6854.613 1393073 PMC1883365

[B39] Rozet I. Metzner J. Brown M. Treggiari M. M. Slimp J. C. Kinney G. (2015). Dexmedetomidine Does Not Affect Evoked Potentials during Spine Surgery. Anesth. Analg 121, 492–501. 10.1213/ANE.0000000000000840 26097987

[B40] Samra M. S. K. Dy E. A. Welch K. B. Ms L. K. Reegept L. Graziano G. P. (2001). Remifentanil- and Fentanyl-Based Anesthesia for Intraoperative Monitoring of Somatosensory Evoked Potentials. Anesth. Analg 92, 1510–1515. 10.1097/00000539-200106000-00031 11375835

[B41] Schwan C. P. Pedersen M. R. Tavanaiepour K. Tavanaiepour D. Hoefnagel A. L. Mongan P. D. (2020). Acute Recurrent Bradycardia with Evoked Potential Loss during Transforaminal Lumbar Interbody Fusion. Anaesth. Rep. 8, 63–66. 10.1002/anr3.12049 33163964 PMC7605407

[B42] Silva-J. M. Jr Katayama H. T. Nogueira F. A. M. Moura T. B. Alves T. L. de Oliveira B. W. (2019). Comparison of Dexmedetomidine and Benzodiazepine for Intraoperative Sedation in Elderly Patients: a Randomized Clinical Trial. Reg. Anesth. Pain Med. 44, 319–324. 10.1136/rapm-2018-100120 30777901

[B43] Stokes S. M. Wakeam E. Antonoff M. B. Backhus L. M. Meguid R. A. Odell D. (2019). Optimizing Health before Elective Thoracic Surgery: Systematic Review of Modifiable Risk Factors and Opportunities for Health Services Research. J. Thorac. Dis. 11, S537–S554. 10.21037/jtd.2019.01.06 31032072 PMC6465421

[B44] SudIvya Sharma P. J. (2013). Dexmedetomidine and Anesthesia. Indian J. Clin. Pract. 24, 223–225.

[B45] Tobias J. D. Goble T. J. Bates G. Anderson J. T. Hoernschemeyer D. G. (2008). Effects of Dexmedetomidine on Intraoperative Motor and Somatosensory Evoked Potential Monitoring during Spinal Surgery in Adolescents. Paediatr. Anaesth. 18, 1082–1088. 10.1111/j.1460-9592.2008.02733.x 18717802

[B46] Tun Liu B. D. Qi H. Yan L. Zhao S. Liu Z. Liu X. (2021). The Prognostic Value of Intraoperative Neuromonitoring by Combining Somatosensoryand Motor-Evoked Potentials for Thoracic Spinal Decompression Surgery in Patients with Neurological Deficit. Spine 54, 25–33. 10.1097/brs.0000000000003989 34435985

[B47] Vitale M. G. Skaggs D. L. Pace G. I. Wright M. L. Matsumoto H. Anderson R. C. (2014). Best Practices in Intraoperative Neuromonitoring in Spine Deformity Surgery: Development of an Intraoperative Checklist to Optimize Response. Spine Deform 2, 333–339. 10.1016/j.jspd.2014.05.003 27927330

[B48] Wang S. Yang Y. Zhang J. Tian Y. Shen J. Wang S. (2017). Frequent Neuromonitoring Loss during the Completion of Vertebral Column Resections in Severe Spinal Deformity Surgery. Spine J. 17, 76–80. 10.1016/j.spinee.2016.08.002 27497889

[B49] Yan Lu E. R. P. (2007). Selective Action of Noradrenaline and Serotonin on Neurones of the Spinal Superficial Dorsal Horn in the Rat. J. Physiol. 582, 127–136. 10.1113/jphysiol.2007.131565 17463043 PMC2075283

[B50] Yoshida G. Ando M. Imagama S. Kawabata S. Yamada K. Kanchiku T. (1976b). Alert Timing and Corresponding Intervention with Intraoperative Spinal Cord Monitoring for High-Risk Spinal Surgery. Spine (Phila Pa. 44, E470–E479. 10.1097/BRS.0000000000002900 30312271

[B51] Yoshida G. Imagama S. Kawabata S. Yamada K. Kanchiku T. Fujiwara Y. (1976a). Adverse Events Related to Transcranial Electric Stimulation for Motor-Evoked Potential Monitoring in High-Risk Spinal Surgery. Spine (Phila Pa. 44, 1435–1440. 10.1097/BRS.0000000000003115 31589200

[B52] Yusuke Funai A. B. Anthony E. (2014). Pickering C, Daisuke Uta a, Kiyonobu Nishikawa B, Takashi Mori B, Akira Asada B, Keiji Imoto A,d, Hidemasa Furue A,d,⇑, Systemic Dexmedetomidine Augments Inhibitory Synaptic Transmission in the Superficial Dorsal Horn through Activation of Descending Noradrenergic Control: An *In Vivo* Patch-Clamp Analysis of Analgesic Mechanisms. PAIN 115, 617–628. 10.1016/j.pain.2013.12.018 PMC4237836 24355412

[B53] Zentner J. Albrecht T. Heuser D. (1992). Influence of Halothane, Enflurane, and Isoflurane on Motor Evoked Potentials. Neurosurgery 31, 298–305. 10.1227/00006123-199208000-00015 1513434

[B54] Zentner J. Thees C. Pechstein U. Scheufler K. M. Würker J. Nadstawek J. (1976). Influence of Nitrous Oxide on Motor-Evoked Potentials. Spine (Phila Pa. 22, 1002–1006. 10.1097/00007632-199705010-00012 9152450

[B55] Zhuang Q. Wang S. Zhang J. Zhao H. Wang Y. Tian Y. (1976). How to Make the Best Use of Intraoperative Motor Evoked Potential Monitoring? Experience in 1162 Consecutive Spinal Deformity Surgical Procedures. Spine (Phila Pa. 39, E1425–E1432. 10.1097/BRS.0000000000000589 25387144

